# Pars Plana Vitrectomy and the Risk of Ocular Hypertension and Glaucoma: Where Are We?

**DOI:** 10.3390/jcm9123994

**Published:** 2020-12-10

**Authors:** Tommaso Rossi, Guido Ripandelli

**Affiliations:** 1 IRCCS Policlinico San Martino, 16100 Genoa, Italy; 2 IRCCS—Fondazione G.B. Bietti ONLUS, 00100 Rome, Italy; guido.ripandelli@fondazionebietti.it

**Keywords:** ascorbate, pars plana vitrectomy, glaucoma, ocular hypertension, oxidative stress, vitreous metabolism

## Abstract

Purpose is to review the pathogenic mechanism for ocular hypertension and glaucoma development after pars plana vitrectomy. Both acute and chronic causes are considered, and special attention is paid to the theories and clinical evidence on the risk of developing Open Angle Glaucoma (OAG) after Pars Plana Vitrectomy (PPV). Most existing scientific literature on the issue agree on the role of ascorbate as an oxygen scavenger within the vitreous chamber. Oxygen tension in the vitreous and anterior chamber is maximum inn proximity of the retinal surface and endothelium, respectively and steeply decreases toward the lens, on both sides, and trabecular meshwork. Vitreous removal and, to a lesser extent, liquefaction, greatly reduces oxygen tension gradient in vitreous chamber while cataract extraction has similar effects on anterior chamber oxygen gradients. Oxygen derivatives originated from the cornea and retina are actively reduced by the vitreous gel and/or the crystalline lens. Vitreous removal and cataract extraction reduce drastically this function. Most reported clinical series confirm this hypothesis although protocol difference and follow-up length greatly impact the reliability of results.

## 1. Introduction

The vitreous gel constitutes the largest part of ocular volume and mass, filling the vitreous chamber comprised between the lens, ciliary body and the retina. The vitreous has been lengthy and erroneously believed to be of little importance for the physiologic functioning of the eye since its removal allowed apparently unaltered vision, to the point that a classic textbook of ophthalmology refers to is stating: “[…] *apart from its role in oculogenesis, the vitreous has no well substantiated function so that an eye devoid of gel is not adversely affected* […]” [1].

Today hundreds of biochemical and metabolic functions are ascribed with certainty to the vitreous that yet still represents a mysterious tissue basilar to ocular development during the fetal life and whose alteration secondary to aging and/or disease may profoundly affect ocular function. The vitreous proteome indeed is acquiring increasing importance as more information regarding its component in normal and pathologic states gets unraveled. Even neurodegenerative diseases may be heralded by specific proteins in the vitreous [2] and research in this subspecialty is thriving.

The vitreous indeed plays a pivotal role in the anatomy and physiology of the eye [3], acting as a scaffold, energy damper [4], nutrients distributor [5], oxygen scavenger and more [6]. Vitreous alterations have been related to a wide spectrum of ocular diseases including retinal tears and detachment, peripheral retinal tears, vitreo-retinal interface syndromes and macular holes, cataract, age related macular degeneration and also many different forms of glaucoma [7].

Pars plana vitrectomy (PPV) is a common surgical procedure aimed at removing the vitreous gel, performed for a variety of indications [8] including retinal detachment, macular puckers and holes, diabetic retinopathy and trauma [9]. Removing the vitreous gel and replacing it with a variety of substances denominated tamponades and including gases, silicone oil and perfluorocarbon liquids, or simply leaving aqueous within the vitreous chamber has shown the potential to significantly alter ocular pressure both acutely and chronically, leading to different forms of ocular hypertension and glaucoma.

Vitreous removal and replacement, in fact, may cause significant intraocular pressure (IOP) increase and glaucoma by means of different mechanisms including tamponade hyper-filling, aqueous misdirection, lens diaphragm shifting, zonular weakness, ciliary body edema, trabecular meshwork damage, vortex vein damage, neovascular stimulation [10] only to name the most frequent ones (Table 1).

Moreover, vitreous removal and its replacement with gas or silicone oil induce a myriad of changes even from the bio-mechanical standpoint and may alter the measure of intraocular pressure itself, compromising our capability to understand fluid behavior and balance within the eye [11]: particularly high viscosity silicone oils may significantly impact assumptions made for ocular applanation tonometry measures and accuracy is impacted [12]. This is secondary to the IOP measure methodology that relies on assumed corneal thickness and, more importantly, corneal stiffness that most likely is affected by dramatic changes in ocular content physical properties such those induced by the presence of tamponade agents.

We will briefly illustrate the most prevalent and important acute and chronic mechanisms for IOP increase both in the short and long term and will focus on what is possibly the most interesting and misunderstood: open angle glaucoma occurring years after uncomplicated pars plana vitrectomy without recurring to long last tamponades and even when no tamponade at all has been used. Interestingly, although the potential for PPV to alter ocular pressure homeostasis had been hypothesized in the very early stages of vitrectomy development [13] the full pathogenic mechanism has not been proposed until much later [14].

The role of PPV in the development of successive open angle glaucoma (OAG) up to several years after gel removal has been lengthy debated in the past years as clinical the proposed pathogenic mechanism gained acceptance and clinical series have been published.

### 1.1. Acute Ocular Hypertension Mechanisms

Acute peri- or post-operative IOP rise recognizes mechanism related to surgery, inflammation and/or tamponade use and their physical and chemical properties (see Table 1).

The risk for optic nerve and retinal damage related to intraoperative ocular pressure correlated to pars plana vitrectomy start from the very beginning as is nowadays understood that intraoperative IOP management is as important as long-term control. IOP peak during surgery may in fact result in long-term damage to the optic nerve and many other ocular structures [15].

Intraocular pressure (IOP) increase over 30 mmHg has been found in over 43% of all eyes undergoing uncomplicated PPV [16] and some authors suggest ever higher figures in the first post-operative week then normalizing spontaneously [17]. Ciliary body inflammation and fibrin formation within the anterior chamber have been regarded as responsible for most transient and acute increase in IOP, often requiring limited or no therapy.

Other frequent mechanisms of acute IOP rise include anterior shifting of the iris-lenticular diaphragm, more often in phakic eyes, especially highly myopic eyes due to weakness of the zonular ligament that allows forward movement of the entire crystalline lens under the buoyancy force of both silicone oil and gas bubbles used as tamponades. Effective treatment for such conditions often is based on limiting unnecessary surgical maneuvers to minimize inflammation, and posturing. In case of iris and lens anterior shifting, the suspicion index must be particularly high and in case posturing fails evaluate the need for making the patient pseudo-phakic as soon as possible since malignant glaucoma mechanism may ensue.

Post-operative choroidal hemorrhage, especially if annular and extended 360° around may be another cause for anterior shifting of the ciliary body, iris and lens and drainage may be necessary to re-establish a deep anterior chamber [18].

Aqueous misdirection syndrome is a well-known cause of acute post-operative IOP increase in phakic eyes undergoing many different types of surgery, mostly trabeculectomy. The same pathogenic mechanism has also been observed in phakic eyes following pars plana vitrectomy [19] as aqueous fails to enter the posterior and the anterior chamber to access the trabecular meshwork and accumulate posterior to the tamponade agent bubble thus increasing posterior to anterior force, shallow the anterior chamber and increase the ocular pressure through an angle closure mechanism giving place to a vicious cycle. Inferior YAG laser iridectomy is usually difficult to make in such conditions and often ineffective so that lens extraction better if associated to surgical Ando’s iridectomy becomes necessary [20].

Excessive filling of the vitreous chamber with silicone oil at the time of fluid injection may also determine acute intraocular pressure rise after surgery, as well as expansile gas injection dilation. Inaccurate estimate of tamponade gas mixtures (SF_6_, C_2_F_6_, C_3_F_8_) may lead to expansile concentrations leading to acute, often disastrous, IOP rise that may result in ophthalmic artery occlusion [21].

Similarly, an acute IOP rise may be due to gas expansion in case of gas anesthesia in patients with gas tamponade and altitude travelling [22] with different mechanisms: in the first case mostly nitrous oxygen [23] migrates from the blood stream into the intraocular gas bubble as a function of its diffusion and partial tension, changing the gas bubble composition; in the second case the same gas bubble exposed to lesser pressure for example at high altitudes or during airplane travelling [24], expands, exerting pressure on the eyewall, leading to pain and possible vascular accidents.

Those occurrences are particularly dangerous as the IOP rise may compromise acutely central retinal artery perfusion and determine retinal and optic nerve ischemic damage similarly to infarction. Prevention is the only effective treatment for such condition and particular attention should be paid to inform patients appropriately on the mandatory prohibition to fly, travel to altitudes, even relatively low rise mountains as car driving would bring the patient to lower atmospheric pressure in relatively short time. Anesthesiologists should always be aware of the presence of gas bubbles in the eye of patients undergoing surgery for any reason, especially non ophthalmic subsequent procedure when the issue is more likely to remain neglected.

A further caveat refers to the notion that intraocular pressure changes also according to head and eye position, as posturing is often required after PPV surgery: IOP in the most dependent eye increases significantly after posturing [25]. This assumes importance in spine surgery for example, when the patient may be postured with tamponated eye in a dependent position, often face down or on a lateral decubitus occasionally reported as cause of ischemic optic nerve damage [26].

### 1.2. Chronic Causes of Ocular Hypertension and Glaucoma

Chronic IOP elevation and glaucomatous damage after pars plana vitrectomy mostly refers to the development of open angle glaucoma although prolonged inflammation may sparkle completely different pathogenic mechanisms: the formation of synechiae in the angle and the ingrowth of neovascularization from the angle and iris, giving rise to completely different types of glaucoma that require specific treatment both pharmacological and surgical, if necessary. Those eyes frequently have a history of recrudescent inflammation and never completely quiet, despite therapy, until overt glaucoma ensues (see Table 1).

More subtle forms of chronic open angle glaucoma after pars plana vitrectomy are related to what is today known as the “the oxidative theory”, a complex sequence of additive biochemical events leading to trabecular meshwork damage. We will focus on this very mechanism since it applies to “uncomplicated” surgery for a completely different indication, often macular pucker or holes that eventually, after years, tend to develop OAG with a higher prevalence compared to fellow-eyes. 

In case of monolateral OAG development after successful pars plana vitrectomy for retinal detachment [9] and silicone oil tamponade, at least four different mechanism leading to glaucoma have been proposed [27] but more notably, the inevitable presence of silicone oil emulsion droplets of microscopic dimension (less than 1 micron [28]) and their demonstrated phagocytosis by means of macrophages and other cells has been demonstrated in virtually all ocular tissues including the trabecular meshwork [29], triggering inflammation and altering its function. 

## 2. Vitreous Physiology and Pathogenic Changes related to Ageing and Pars Plana Vitrectomy

Understanding the oxygen derivatives metabolism within the vitreous chamber will help understand the extremely delicate cohabitation and balance between tissues laying on opposite sides of the vitreous chamber and yet at the extremities of metabolic oxygen consumption and oxygen-derivatives damage susceptibility: the retina, lens and trabecular meshwork.

The retina, in fact, is one of the highest oxygen intermediates producer per weight unit in the whole human body [30] while the lens and trabecular meshwork need a low oxygen tension to function properly. The vitreous gel is therefore strategically positioned in between such structures with diametrically opposed metabolic needs and its removal or alteration leads to dramatic changes that deeply impact crystalline lens and trabecular meshwork functions.

Among the many and increasing number of functions and biochemical activities attributed to the vitreous, the role of ascorbate certainly plays a prominent role. Ascorbate blood concentration is 50–60 μM while is 30–40 times higher in the human vitreous (about 2 mM) [31] where is actively transported by a sodium-dependent transporter named SLC23A2 present in the ciliary epithelium pigmented layer [32].

Why is so much ascorbate needed within the vitreous chamber? Shui et al. [3] proposed that intraocular oxygen tension is regulated in an ascorbate-dependent way: oxygen reacts with ascorbate to produce hydrogen peroxide, then converted to H_2_O by the action of catalase. In this scenario, the vitreous would act as a barrier to oxygen derivates strategically interposed between the highly vascularized and metabolically active retina and the delicate anterior structures extremely sensitive to oxidative stress: the lens and trabecular meshwork meshwork. 

Siegfried et al. [33] confirmed Chang’s hypothesis of an oxidative stress damage to the lens and trabecular meshwork at the basis of both cataract formation and glaucoma development thereafter by measuring oxygen tension in the anterior chamber eyes undergoing anterior segment surgery and in the vitreous chamber of eyes undergoing vitrectomy before and after vitreous gel removal. They noted a steep gradient of oxygen tension both in the anterior chamber and vitreous chamber decreasing towards the lens and trabecular meshwork until the vitreous and crystalline lens are present; after their removal that gradient disappears and oxygen tension almost equals anywhere within the eye, showing a significant increase at the trabecular meshwork and lens. 

Shui et al. also noticed that the liquified vitreous contains less ascorbate and consumes oxygen at a much lower rate than vitreous gel [3]. Until the vitreous is mainly in a gel status, oxygen diffusion from the retina encounters ascorbate and gets consumed as witnessed by the decreasing oxygen gradient as the distance from the retina increases [14,33]. When the vitreous liquefies or is replaced by aqueous as after PPV, oxygen moves transported by aqueous currents and turbulence generated by saccades and head motion [15] reaching comparable concentration throughout the eye. Filas et al. demonstrated through computational fluid dynamics [34] that increasing vitreous velocity and or oxygen diffusion, both factors greatly contribute to the abatement of oxygen tension gradient physiologically present from the retina to the lens.

This may suggest a role of vitreous liquefaction in the development of nuclear cataract, as liquified gel current increase oxygen levels close to the lens altering the physiologic gradient acting as consecutive barriers protecting the crystalline transparency. Oxygen and oxidative stress are well-known risk factors for cataract development. Oxygen has been linked to apoptosis in trabecular meshwork cells [35] which would represent the very last piece of the pathogenic mechanism puzzle although the same reasoning brings to believe vitrectomy might be beneficial for ischemic retinal diseases.

## 3. Clinical Evidence

The very first to report OAG increase after PPV were probably Stangos et al., in 2004 [36] who observed OAG doubling (from 19.7% to 38%) nine months after PPV although they did not comment this finding that was later on underlined by Chang in his 2006 Jackson Lecture when he presented data related to 453 eyes followed for an average 56.9 months and proposed the oxidative theory. He also pointed out that phakic eyes had a significantly longer time between PPV and glaucoma diagnosis compared to pseudo-phakic patients as if the lens acted as oxygen scavenger itself while developing a cataract. 

Luk et al. [37] confirmed this hypothesis having followed 101 patients for an average 51 months after PPV for macular surgery and found a 7.9% prevalence of OAG with a significant difference between phakic (2%) and pseudo-phakic (13%).

Not all data concurred: Yu et al. in 2010 [38] retrospectively reviewed the records of 441 patients followed for an average 79 months and found 4.31% OAG after PPV versus 2.49% in the controls concluding the difference was not statistically significant, in agreement with Mi et al. who in 2015 [39] retrospectively analyzed 234 eyes after epiretinal membrane peeling and PPV at least 2 years after surgery and found no evidence of increased OAG prevalence.

Koreen et al. [40] in 2012 followed 286 eyes with more than 6 months follow-up after PPV and found an overall 11.6% of OAG with highly statistically significant difference between phakic patients 1.4% and pseudo-phakic patients (16%). In that very series there was no difference between those who underwent cataract extraction before, at the time of PPV or later on, therefore the lens itself proved a protective factor against the onset of OAG.

Govetto et al. [41] in 2014 also found a significant difference in the rate of OAG of 312 eyes (8.9% of vitrectomized vs. 2% of non-vitrectomized eyes) between 3 and 6 years after PPV while Fujikawa [42] found an increase in intraocular pressure after PPV for macular hole but not for epiretinal membrane (ERM) 12 months after surgery, possibly due to the shortness of follow-up or the less complete vitrectomy usually performed in ERM cases. It is conceivable that if a significant amount of peripheral vitreous is left in place, as is the case for many surgeons performing macular surgery, this allows a residual oxygen-binding effect postponing the insurgence of cataract and trabecular meshwork damage.

Yamamoto [43] found a significant increase in IOP only for patients undergoing PPV for retinal detachment compared to MH and ERM who did not show such an increase after an average 23 months follow-up and the PROVE study [44] demonstrated a significant increase in IOP and decrease in OCT measured retinal nerve fiber layer (RNFL) thickness 12 months after PPV for macular surgery although the shortness of follow-up and peeling maneuvers may act as confounders.

Miele at al. in 2018 [45] conducted a meta-analysis on the issue, pooling seven paired studies of cases versus controls and a mean follow-up of 12 months. Only four studies reported OAG data on 851 patients and the prevalence were 7.8% among PPV patients and 4.8% of controls, with a 1.67 odds ratio. Ocular hypertension was found in 5.8% and 3.1% of patients, respectively. The study concluded there is an increase in the risk for OAG after PPV, but protocol inconsistency prevented conclusive evidence with available data.

Mansukhani et al. [46] also reported a surgical series of patients undergoing retinal surgery, operated on for scleral buckling (SB) alone, PPV and PPV associated to SB, with 10 years follow-up. Interestingly, the 10 years cumulative risk of developing OAG was 0 in the SB group, 17% in PPV group and 10% in the combined PPV and SB group.

## 4. Discussion and Conclusions

Ocular pressure homeostasis is a precious and delicate equilibrium actively pursued through a number of precisely tuned mechanism and vitreous removal is a gross maneuver, literally an amputation that cannot but have profound consequences on many of those intricate metabolisms.

The prevention and management of intraocular pressure alterations related to pars plana is a multifaceted and articulate issue: dealing with acute ocular hypertension is more straightforward and usually effective. For what concerns the development of open angle glaucoma with or without silicone oil emulsion the problem is virtually ineludible and intrinsic to the surgical procedure.

Although the seeds of potential chronic harm secondary to vitreous removal were planted at the very beginning of the vitrectomy era, the lengthy latency and complex pathogenesis delayed the acquisition of full consciousness until much later on.

From the very moment a vitrectomy starts, the surgeon takes a somewhat illusory control of intraocular pressure but must pay extreme attention to leave it as much unaltered as possible. When surgery ends, the injection of tamponades, both gas and liquids will inevitably have marked repercussions on pressure short and long-term control: oil emulsification leads to macrophage and monocyte phagocytosis and activation and even leaving aqueous in the vitreous chamber will forever destroy the ascorbate-based oxygen scavenging mechanism. This will de facto open the door to future inevitable events such as progressive and consecutive crystalline lens and trabecular meshwork meshwork oxidation as a ticking bomb. This unless game-changer vitreous substitutes appear at the horizon.

In the meantime it is imperative to counsel post pars plana vitrectomy patients appropriately as to the risk of developing secondary (should we call it) open angle glaucoma even years after the surgical procedure and more likely so after cataract removal if performed at a later time. For many surgeons used to combine phacoemulsification to pars plana vitrectomy almost irrespective of patients age, or at least in patients in their fifties or early sixties it may be wise to reconsider this approach until an overt cataractous lens develops as this is actually protecting the trabecular meshwork from a much less repairable damage. 

It is unclear at the moment if the subgroup of patients at risk for glaucoma for many reasons and glaucoma suspects are more prone than the remainder of the population to develop post pars plana vitrectomy open angle glaucoma but it seems likely and wise to suspect it and behave accordingly.

Most surgical series reported after the enlightening Jackson Lecture given by Stanley Chang on this issue in 2006, proposing the oxidative pathogenic mechanism, have been largely confirming (with a few exceptions) the oxidation theory and the role of the crystalline lens. The only available meta-analysis pointed out the lack of standardization and the need for longer follow-up on similar series in order to be able to shed more light on the issue and acquire solid data that may help us understand the numbers and entity of the problem.

There is today enough convincing experimental evidence to believe that the vitreous gel plays a pivotal role in oxygen gradient maintenance throughout the vitreous chamber and that vitreous liquefaction and more overtly vitreous removal determine an abrupt loss of such gradient. There also is evidence that after vitrectomy the crystalline lens itself, if present, acts as an oxygen scavenger paying the price of speeding the cataract process. After cataract removal in previously vitrectomized eyes, both barriers resisting to oxygen derivatives spreading throughout the eye (i.e., the vitreous and the lens), fail to work and the trabecular meshwork becomes a very likely target for oxidation triggering an intricate metabolic cascade that leads to trabecular cell apoptosis, trabecular function decline and glaucoma. While the role of ocular hypertension in causing optic nerve head hypoperfusion thus promoting oxidative stress derived damage in ganglion cell axons is known [47], at the time being we can only speculate on the possible role of vitreous removal in promoting also a similar mechanism.

Effective countermeasures are difficult to imagine at the time being, except for the development of an entirely new generation of vitreous substitutes capable of keeping the oxygen gradient within the vitreous chamber, something difficult even to imagine with the present level of insight on this specific topic. 

Biomimetic gels with tunable mechanical properties have been developed with lengthy research [48] and the most promising at present are possibly those injected as a double liquid component that gel at 37° within the eye and have been considered safe for porcine retina up to 4 weeks before undergoing liquefaction. The many difficulties encountered in decades of vitreous substitute research and the promising results [49] yet incomparable to the magnificent and amazing physical, chemical, optical, biological and time-enduring qualities of the human vitreous increase, if possible, the wonder of even life-long students for this pristine and mysterious tissue.

All tested hydrogels, no matter how promising, may mimic the ascorbate system keeping the oxygen gradient within the vitreous chamber. For this reason and possibly for a long time still to come, the risk of OAG should be overtly discussed with patients especially because PPV indications since the very first cases from Machemer [50] have nowadays expanded to include less invalidating conditions such as vitreous floaters. The risk to benefit ratio for every single procedure will need careful consideration and thorough patients’ comprehension as Hippocrates himself thought us.

## Figures and Tables

**Table 1:** Most frequent types and mechanism of intraocular pressure rise after pars plana vitrectomy.

Type	Mechanism	Acute vs. Chronic
SiO Hyperfilling	Vitreous chamber	Acute
Aqueous Misdirection	Malignant glaucoma—posterior aqueous misdirection	Acute
Iris-Lens diaphragm shifting	Cicliary body edema, choroidal detachment	Acute
AC Inflammation	Presence of fibrin/blood/trabecular meshwork edema	Acute
Gas expansion	Air or high altitude travelling/gas anesthesia	Acute
Posturing	Spine surgery with lateral of prone decubitus	Acute
Angle synechiae	Chronic inflammation in the AC, intermittent closure	Chronic
Neovascularization	Secondary to anterior segment ischemia	Chronic
SiO Glaucoma	SiO emulsion droplets block phagocytosis and are phagocytosed by trabeculocytes	Chronic
Open Angle Glaucoma	oxidative stress increases trabecular resistance	Chronic
